# Veterinarians Needed in the United States Army

**Published:** 1901-10

**Authors:** 


### VETERINARIANS NEEDED IN THE UNITED STATES ARMY.

There are now twenty-one vacancies in the office of veterinarian, United States army, and four more will occur in the near future. Veterinarians are required for service in the cavalry and field artillery. They are allowed the pay and allowance of a second lieutenant of cavalry—$1500 per annum—with 10 per cent. increase for each period of five years' service up to twenty years, and quarters. They are not commissioned officers, but wear the uniform of a second lieutenant, without the shoulder-straps.

It is the intention of the War Department to hold a competitive examination at an early date for the purpose of filling the vacant places, and anyone desiring to take the examination should make application at once to the Adjutant-General of the Army, Washington, D. C.

Applicants must be citizens of the United States, not less than twenty-two nor more than thirty-five years of age; must be graduates of some recognized veterinary college, and must be of good moral character and physically sound. They will be required to pass a written examination in the following subjects:

*Basis examination.* English grammar, arithmetic, geography, history.

*Subject examination.* Anatomy and physiology, pathology, practice of medicine, descriptive and operative surgery, materia medica and therapeutics, sanitary medicine, conformation of the horse, and examination for soundness, horse-shoeing, and meat-inspection. Veterinary hygiene: General feeding and watering, stabling and care of animals in garrison and field, saddling, bitting, packing, etc.

---

National Horse-show Association of America. The prize list for the Seventeenth Annual Exhibition of the National Horse-show Association of America, to be held at Madison Square Garden, New York, November 18 to 23, 1901, announces $30,000 in premiums. Entries close Saturday, October 26, 1901. Address the Secretary, John G. Hecksher, Esq., 16 East 23d Street, New York City.

